# Retrospective Evaluation of the Use and Complications of Small-Bore Wire-Guided Thoracostomy Tubes in Dogs and Cats: 156 Cases (2007–2019)

**DOI:** 10.3389/fvets.2022.818055

**Published:** 2022-03-31

**Authors:** Tomas Boullhesen Williams, Daniel Fletcher, Jacqueline Fusco, Allison Bichoupan, Lisa Weikert, Mario Barenas, Julie Menard

**Affiliations:** ^1^Department of Clinical Sciences, College of Veterinary Medicine, Cornell University, Ithaca, NY, United States; ^2^Department of Veterinary Clinical and Diagnostic Sciences, Faculty of Veterinary Medicine, University of Calgary, Calgary, AB, Canada

**Keywords:** pneumothorax, pleural space disease, pyothorax, Seldinger, chest tube

## Abstract

**Background:**

Small-bore wire-guided thoracostomy tubes (SBWGTT) are commonly used in small animals for management of pleural space disease. We aimed to evaluate the indications, placement locations, types of complications, and complication rate of small-bore wire-guided thoracostomy tube placements in dogs and cats in a university setting.

**Methods:**

Electronic medical records of patients that underwent SBWGTT placement were reviewed. Signalment, disease, outcome, indication for thoracostomy tube, placement location, number of attempts, diagnostic imaging, number, and type (insertional, technical, and infectious) of complications were recorded. Logistic regression analysis was performed to determine risk factors for complications.

**Results:**

A hundred fifty-six cases were identified between 2007 and 2019. Traumatic pneumothorax (33%), pyothorax (25%), and spontaneous pneumothorax (16%) were the most common indications for placement of a SBWGTT. Complications developed in 50 cases (32%). Technical and insertional complications accounted for 21.7% and 14.1% of all cases. Infectious complications were rare with 3.1% of all cases. Pneumothorax (19%), soft tissue swelling at insertion site (14%), and kinking of the chest tube (13%) were most common. Accidental lung perforation was reported in 5/50 complications (7%). Multiple chest tube placement attempts were associated with complications (OR = 6.01 CI: 2.13 to 16.93 *p* = 0.0007).

**Conclusions:**

Complications of SBWGTT placement occurred in one third of cases. Serious complications such as accidental lung perforation was reported in two cases. Complications were associated with number of attempts.

## Introduction

Thoracostomy tubes are commonly used in small animals for the management of pleural space disease, either prophylactically following thoracotomy or therapeutically. Therapeutic thoracostomy chest tube placement is usually indicated when multiple needle thoracenteses are required to alleviate the patient's respiratory compromise due to accumulation of fluid or air within the pleural space. Thoracocentesis is associated with some complications, and chest tube placement may be advantageous for patient comfort or decreased sedation/anesthesia risks (1–3). In veterinary medicine, the most commonly used thoracostomy tubes include the traditional trocar type, small-bore wire-guided thoracostomy tubes (SBWGTT), and recently Jackson Pratt thoracostomy drains (4–6). The larger-bore trocar types have traditionally been used for the drainage of dense and thick material from the pleural space (7). Placement requires general anesthesia, and substantial training is required prior to first insertion. Their use is associated with complication rates in humans ranging from 5 to 35% (8–10) and from 44 to 58% in veterinary patients (6, 11–13). Reported complications in veterinary patients include vessel laceration, pneumothorax, lung puncture, pain, fluid leakage from insertion site, and failure to drain due to tube failure (6, 11–14). For the past decade, SBWGTT have gained in popularity as they are easier to place, do not require general anesthesia, and are thought to be less painful. Complications are reportedly rare with obstruction, mispositioning, kinking, and accidental removal by the animal being described (6, 15). In human medicine, there is controversy about the use of small-bore chest tubes for treatment of pyothoraces with the concern that highly viscous fluid could clog the tube (8, 9, 16). However, a canine cadaver study showed no difference in removal of air or low- or high-viscosity fluid between large- and small-bore thoracostomy tubes (17). This further confirms clinical reports of successful management of pyothorax in dogs and cats with SBWGTT (7, 15). Despite the wide use of SBWGTT in dogs and cats for the last decade, limited information exists about associated indications and complications. Our goal was to evaluate the indications, placement locations, types of complications, and complication rate for SBWGTT placements in dogs and cats in a university setting.

## Materials and Methods

### Data Collection

This was a retrospective observational study. Electronic medical records (EMR) were searched for billing fees for SBWGTT and/or thoracostomy placement between January 1, 2007, and December 31, 2019, and reviewed. SBWGTT were introduced to our tertiary referral teaching hospital in 2007. Only SBWGTT placed percutaneously using the modified Seldinger technique were included; SBWGTT chest tubes placed intraoperatively were excluded. Data, including signalment, disease, outcome, indication for thoracostomy tube, placement location, number of attempts, use of sedation or general anesthesia for placement, diagnostic imaging performed to assess chest tube placement, number and type of complications, and clinician involved in placement, were recorded (JF, AB, LW, MB) and then reviewed by a single author for uniformity (TBW). Outcome was recorded as alive at discharge, died, or euthanized. Clinicians involved in placement were categorized into two groups: emergency and critical care (ECC) clinician (specialty ECC intern, ECC resident, or ECC faculty), and non-ECC clinician (all other clinicians, including small animal rotating interns and residents from the following specialties: anesthesia, cardiology, internal medicine, surgery, and oncology).

**Figure 1:**
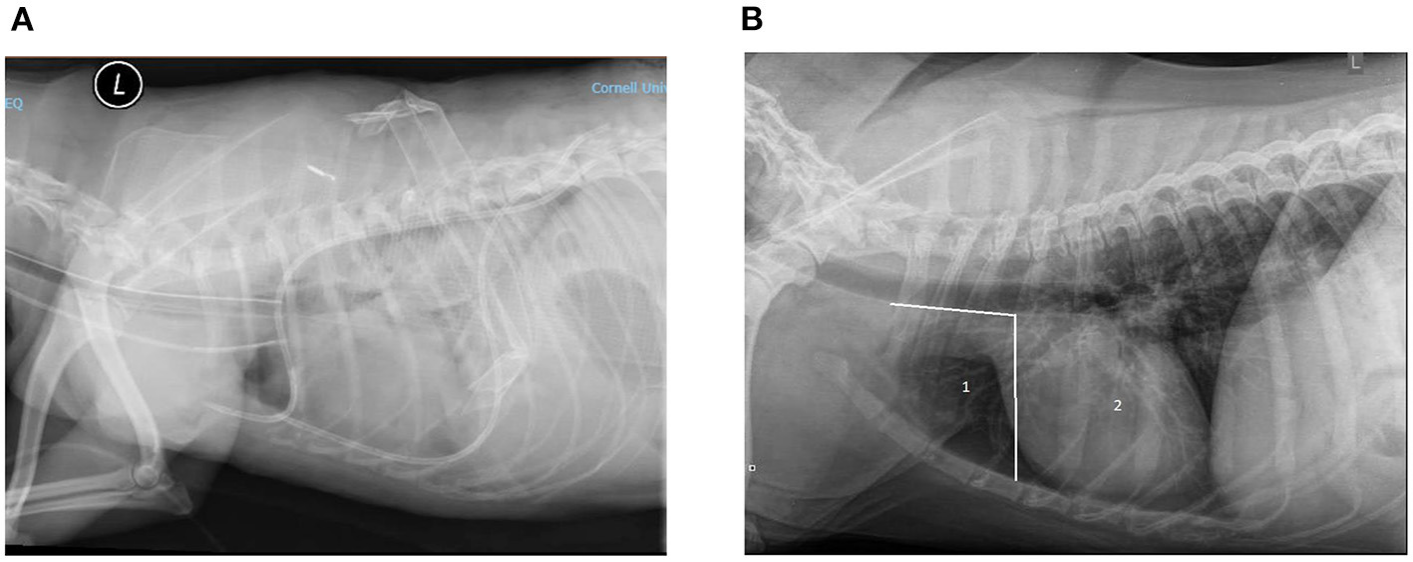
**(A)** Example of ideal chest tube placement. Left lateral radiograph of a canine thorax showing the presence of two SBWGTT in the pleural space. This patient was under anesthesia and intubated; an endotracheal tube can be seen in the trachea. A radio-opaque catheter can been seen in the jugular. Increased soft tissue opacity of the pulmonary field and pleural effusion are also seen. **(B)** Assessment of SBWGTT placement accuracy. Zone 1 or ideal placement zone: between cranial-most aspect of the thoracic cavity and caudal edge of the third rib, ventral to the trachea. Zone 2: not ideal placement zone from the caudal edge of third rib to the diaphragm.

Diagnostic imaging reports were assessed for evidence of complications. Reports from thoracic radiographs and computed tomography scans were reviewed for mention of complications associated with thoracostomy tube placement. In addition, when available, images were reviewed by TBW for assessment of accuracy in thoracostomy tube placement. Appropriate thoracostomy tube placement was determined based on anatomical positioning of the chest tube as depicted in Figure 1. Ideal thoracostomy tube placement was defined as placement of the tip of the chest tube cranial to the third intercostal space and ventral to the trachea. Bilateral chest tube placements were considered appropriate only if both were placed correctly.

The number of placement attempts was recorded for each patient. Each placement was considered one attempt; therefore, animals with bilateral thoracostomy tubes were noted to have a minimum of two attempts. Complications were recorded and assigned to one of the three following categories: insertional, technical, or infectious. Insertional complications include intraparenchymal lung placement, chest wall trauma, pneumothorax, and pneumomediastinum. Technical complications include thoracostomy tubes kinking, accidental disconnection or dislodgement, tube leakage, tube migration, subcutaneous emphysema, and soft tissue swelling at the insertion site. Infectious complications include discharge, abscess, or cellulitis at the insertion site.

The same brand of SBWGTT was used for the duration of the study (MILA International, Inc. guidewire inserted chest tube 14 and 12 Ga). Indications, need for sedation, local and/or general anesthesia, placement technique (including type of suture and pattern used to secure the tubes), diagnostic imaging test, and patient management (including use of bandage over the SBWGTT, medications, frequency of SBWGTT checks and drainage) were at the attending clinician's discretion. Placement technique was not standardized, and variations may have occurred. In addition, a dilator was added to the guidewire insertion kit after a few years, and its use was also not standardized or consistently recorded in the EMR.

### Statistical Analysis

Data were assessed for normal distribution with the Shapiro Wilk test. Descriptive statistics (mean and standard deviation for normally distributed data and median and range for data that were not normally distributed) were reported for the following continuous variables: age, body weight, length of hospitalization, and vital parameters (heart rate, respiratory rate, and temperature). Continuous variables were compared between species using unpaired Student's *t*-test or the Mann–Whitney U test. Relative frequencies of categorical data (outcome, indication, placement side, type of imaging modality used to determine placement of the thoracostomy tube, complication type, clinician group involved in placement) were compared using Fisher's exact test or chi-squared test. Complication rate, distribution of complication types, and number of attempts were compared between species using chi-squared likelihood ratio. Complication types (technical, insertional, and infectious) were compared between species and between them using Friedman ANOVA. When more than one type of complication was present, the case was counted in all applicable complication categories. *Post-hoc* Bonferroni corrections were applied to account for multiple comparisons. Logistic regression analysis was performed to determine risk factors for the presence of any complication. The following variables were initially included in the model: species, use of general anesthesia, age at the time of chest tube placement, body weight, indication (pneumothorax vs. others), placement side where three binary variables were used (left, right, and both left and right), and the group of the clinician placing the chest tube (ECC vs. non-ECC). Alpha was set at 0.05. Only variables with *p* < 0.05 were included in the final model. All analyses were conducted using commercial software (IBM Corp. Released 2020. IBM SPSS Statistics for Macintosh, Version 27.0. Armonk, NY: IBM Corp).

Given the retrospective nature of the study, an institutional animal care and use committee waived the need for approval. Owners gave informed consent for placement of thoracostomy tube placement at the time of hospitalization.

## Results

EMRs identified 185 cases with the corresponding billing fee associated with SBWGTT and/or thoracostomy tube placement. Of those, 20 cases were excluded as no records that SBWGTT had been placed were found in the EMR, and nine were excluded because the tubes were placed intraoperatively during thoracic surgery. Following review, 156 cases were available for data analysis.

### Characteristics of Animals With Small Bore Thoracostomy Tube

Thirty-three cats and 123 dogs had SBWGTT placed during the study period. Bilateral tubes were placed in 84/156 cases. Descriptive statistics are shown in Table 1. Naturally, cats were significantly lighter than dogs and had elevated heart rates compared with dogs. There was no difference in complication rate between dogs (31.1%) and cats (36.4%); Chi-squared likelihood ratio *p* = 0.598.

**Table 1:** Case population characteristics.

**Parameter**	**All cases (*n* = 156)**	**Dogs (*n* = 123)**	**Cats (*n* = 33)**	***p*-value**
Age (years)	4 (6.5) (0.08–16)	4.00 (6.650) (0.08–13)	4 (9.5) (0.12–16)	0.21
Body weight (kg)	24.8 (30.37) (2.3–91)	29.8 (18.70) (2.8–91.00)	4.27 (1) (2.3–10)	**<0.001**
Sex (FI/FS/MC/MI)	15/36/83/21	15/27/60/21	0/9/23/0^*^	
Temperature (F)	101.4 (2.8) (92–106)	101.7 (2.10 (97.2–106)	100.7 (3.02) (92.9–104)	0.65
HR (bpm)	149 (60) (72–240)	140 (40) (72–220)	175 (51.5) (120–240)	**<0.001**
RR (bpm)	58 (25)	57(26)	61 (18)	0.66
**Outcome**
Alive *n* (%)	123 (78.8%)	99 (80.5)	24 (72.7)	0.31
Died, *n* (%)	6 (3.8)	4 (3.3%)	2 (6.1)	0.48
Euthanized, *n* (%)	27 (17.3)	20 (16.3)	7 (21.2)	0.48
Length of hospitalization (days)	5 (4) (1–29)	5 (3) (1–29)	4 (6) (1–13)	0.28

* *One cat had unknown sex. Data are presented as mean ± SD for normally distributed data and median (IQR) for non-normally distributed data. FI, female intact; FS, female spayed; MC, male castrated; MI, male intact; HR, heart rate; RR, respiratory rate. The bold value indicates p <0.05*.

### Indications

As described in Table 2, traumatic pneumothorax 51/156 (33%), pyothorax 39/156 (25%), and spontaneous pneumothorax 25/ 156 (16%) were the most common indications for placement of a SBWGTT. Indications for thoracostomy tube placement were significantly different between species (chi-squared likelihood ratio <0.001). The majority of the SBWGTT were placed for management of pyothorax in cats (51.5% in cats vs. 19.5% in dogs, *p* = 0.00022). Pneumothorax was the most frequent indication for placement of SBWGTT in dogs (63.4 vs. 21.2% in cats, *p* = 0.00002). Sedation was used in 88/156 (54%) of the cases, general anesthesia in 45/156 (22%), and a combination of both was used in 12/156 (20%) and did not differ between species. There was no difference with regards to on which side the thoracostomy tubes were placed between species (Pearson chi-squared *p* = 0.568) with the majority of cases (54.9%) having bilateral thoracostomy tubes placed.

**Table 2:** Indications for placement of small-bore guide-wire chest tubes.

**Indication**	**All cases**	**Dogs**	**Cats**	***p*-value**
Chylothorax *n*, (%)	10 (6.4)	6 (4.9)	4 (12.1)	0.13
Congestive heart failure *n*, (%)	1 (0.6)	1 (0.8)	0 (0)	0.62
Hemothorax- trauma *n*, (%)	5 (3.2)	4 (3.3)	1 (3)	0.92
Neoplasia *n*, (%)	6 (3.8)	4 (3.3)	2 (6.1)	0.48
Non cardiogenic effusion *n*, (%)	3 (3.8)	3 (2.4)	0 (0)	0.37
Other *n*, (%)	3 (1.9)	1 (0.8)	2 (6.1)	0.06
Pneumothorax- all *n*, (%)	85 (54.5)	78 (63.4)	7 (21.2)	**<0.001**
Pneumothorax- other causes *n*, (%)	4	3	1	
Pneumothorax spontaneous *n*, (%)	24	24	0	
Pneumothorax trauma *n*, (%)	55	49	6	
Pneumothorax as a complication of Mechanical Ventilation *n*, (%)	2	2	0	
Pyothorax *n*, (%)	41 (26.3)	24 (19.5)	17 (51.5)	**<0.001**
Pulmonary contusions *n*, (%)	2 (1.3)	2 (1.6)	0 (0)	0.48
Total *n* (%)	156 (100)	123 (100)	33 (100)	

*The bold value indicates p <0.05*.

### Placement of Small-Bore Wire-Guided Thoracostomy Tubes

ECC clinicians placed the majority of chest tubes (64% of SBWGTT), but the proportion of type of clinician placing the tubes was not different between species (ECC clinicians placed 63% in dogs vs. 67.7% in cats, chi-squared *p* = 0.62). There was no difference in the number of complications observed between species (chi-squared likelihood ratio *p* = 0.344) as shown in Table 3.

**Table 3:** Characteristics of small-bore guide-wire chest tube placement and complications.

**Parameter**	**All cases (156)**	**Dogs (123)**	**Cats (33)**	***p*-value**
Contention method				0.38
Sedation *n* (%)	88 (60.7)	70 (60.3)	18 (62.1)	0.84
General anesthesia *n* (%)	45 (31)	38 (32.8)	7 (21.1)	0.37
Both *n* (%)	12 (8.3)	8 (6.9)	4 (13.8)	0.23
Placement side (3 unknown side)				0.56
Left *n* (%)	33 (21.6)	27 (22.5)	6 (18.2)	0.62
Right *n* (%)	36 (23.5)	26 (21.7)	10 (30.3)	0.32
Bilateral *n* (%)	84 (54.9)	67 (55.8)	17 (51.5)	0.69
Clinician placing chest tube				Fisher exact test: 0.679
ECC^*^n (%)	96 (64)	75 (63)	21 (67.7)	0.62
Non ECC^*^n (%)	54 (36)	44 (37)	10 (32.3)	0.62
unknown	6	4	2	
Complication present *n* (%)	50 (32.3)	38 (31.1)	12 (36.4)	Fisher exact test:0.675
Complication numbers				
0 *n* (%)	106 (67.9)	85 (69.1)	21 (63.6)	0.55
1 *n* (%)	30 (19.2)	23 (18.7)	7 (21.2)	0.76
2 *n* (%)	15 (9.6)	13 (10.6)	2 (6.1)	0.42
3 *n* (%)	3 (1.9)	1 (0.8)	2 (6.1)	0.06
4 *n* (%)	2 (1.3)	1 (0.8)	1 (3)	0.32
Number of attempts (4 unknown)				0.13
1 *n* (%)	62 (41.9)	49 (41.2)	13 (44.8)	0.69
2 *n* (%)	67 (45.3)	54 (45.4)	13 (44.8)	0.92
3 *n* (%)	16 (10.8)	15 (12.6)	1 (3.4)	0.16
4 *n* (%)	3 (2)	1 (0.8)	2 (6.9)	0.04

* *ECC, emergency and critical care clinician; non-ECC, non-emergency and critical care clinician*.

There was no difference between species in the type of diagnostic imaging performed before and after placement of thoracostomy tubes. Thoracic radiographs were obtained prior to SBWGTT placement in 76/123 (61.8%) dogs and 16/33 (48.5%) cats (Fisher's exact test *p* = 0.231). Following placement of SBWGTT, radiographs were obtained in 97/123 (78.9%) dogs and 24/33 (72.7%) cats (Fisher's exact test *p* = 0.484). Computed tomography (CT) scans were obtained in 58/123 (47.2%) and (10/33) cats (30.3%) (Fisher's exact test *p* = 0.113) and 6/33 (18.2%) cats and 45/123 (36.6%) dogs had both modalities following thoracostomy tube placement (Pearson Chi-squared *p* = 0.131).

**Figure 2:**
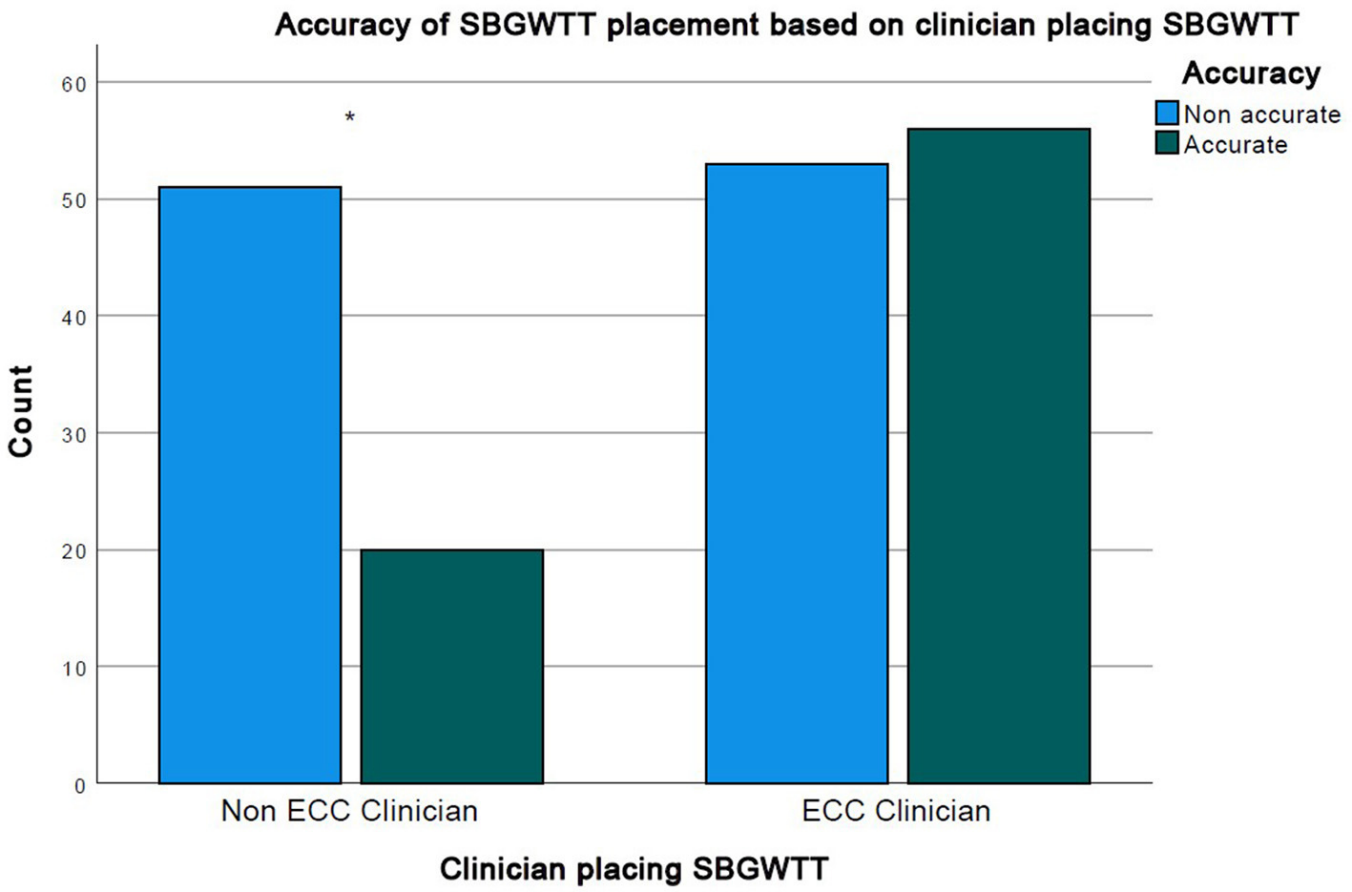
Level of clinician experience vs. accuracy of chest tube placement. *Denotes *p* < 0.005.

Radiographic review of placement accuracy was available in 132 patients with 180 chest tube placements. Ideal placement was achieved in 76/180 (42%) of the placements overall. SBWGTT placed by ECC clinicians were more likely to be accurately placed (56/109 = 51.4%) than SBWGTT placed by non-ECC clinicians (20/71 = 28.2%, chi-squared test *p* = 0.0021) (Figure 2).

Number of placement attempts was recorded in 142 cases with a median of 2 (1–4) attempts per thoracostomy tube placement. There was no difference in the number of attempts per tube between species (chi-squared likelihood ratio *p* = 0.137). Complications developed in 50 cases (32%), with similar frequencies between dogs (38/126 = 31.1%) and cats (12/33 = 36.4%, Fisher's exact test, *p* = 0.675. Overall technical complications (21.7%) were more frequent than insertional complications (14.1%) (Friedman ANOVA with Bonferroni correction *p* = 0.016) and were significantly more frequent than infectious complications (3.1%) (Friedman ANOVA with Bonferroni correction *p* < 0.001). Insertional complications were also more frequent than infectious complications (Friedman ANOVA with Bonferroni correction *p* = 0.002) as described in Table 4. Complications included pneumothorax 13/50 (19%), soft tissue swelling at insertion site 10/50 (14%), kinking of the thoracostomy tubes 9/50 (13%), subcutaneous emphysema 8/50 (12%), disconnection of the tube 8/50 (12%), placement and/or migration into the thoracic wall 4/50 (6%), pulmonary contusions attributed to thoracostomy tube placement 3/50 (4%), discharge at the insertion site 3/50 (4%), lung perforation requiring lobectomy 2/50 (3%), pneumomediastinum 2/50 (3%), leakage of the thoracostomy tubes 2/50 (3%), and others (3%).

**Table 4:** Types of complications of small-bore guide-wire chest tube placement.

	**All cases**	**Dogs**	**Cats**	***p*-value = 0.309**
Insertional *n* (%)	12 (7.7)^*^	10 (8.1)	2 (6.1)	0.68
Technical *n* (%)	25 (16)^∧^	19 (15.4)	6 (18.2)	0.68
Infectious *n* (%)	1 (0.6)	0 (0)	1 (3)	
Insertional + technical *n* (%)	7 (4.5)	5 (4.1)	2 (6.1)	0.61
Insertional + infectious *n* (%)	2 (1.3)	2 (1.6)	0	
Technical + infectious *n* (%)	1 (0.6)	0 (0)	1 (3)	
All 3 *n* (%)^**^	1 (0.6)	1 (0.8)	0 (0)	0.61

* *Significantly different than infectious complications (Friedman Anova with Bonferroni correction p = 0.002),*

∧ *Significantly different than infectious complications Friedman Anova with Bonferroni correction p < 0.001). Statistics were not performed for types of complication with <2 cases.*

** *All three types of complication were present in a single patient*.

Logistic regression analysis showed that of species, use of general anesthesia, age at the time of chest tube placement, body weight, indication (pneumothorax vs. others), placement side, and the type of clinician placing the chest tube (ECC vs. non-ECC), the only risk factors associated with development of a complication was more than two SBWGTT placement attempts (OR = 6.01, 95% CI: 2.13–16.93 *p* = 0.0007).

## Discussion

To our knowledge, this is the first large retrospective review of complications associated with placement of SBWGTT in small animals. Although thought to be safer than trocar-type chest tubes, one third of the cases in this study had some type of complication. Complications are previously reported with intraoperative placement of trocar-type thoracostomy tubes and Jackson-Pratt thoracic drains (6) with leaks and tube breakage being most prevalent. Complications associated with placement of SBWGTT are previously reported in small case series. Similar to the findings in this study, Vatolina et al. (15) describes technical issues, such as kinking, mispositioning, and removal by animals. Those issues may be related to the suturing technique and pattern used to secure the chest tube to the patient. This can cause local irritation due to increased pulling and tugging of the thoracostomy tubes and kinking associated with either position on the chest when not in use, or the weight of the tubing with continuous suction devices attached. The design of specific thoracic shirts to secure tubes close to the body wall may decrease the risk associated with excessive motion and incidence of those complications. The use of an Elizabethan collar is recommended to decrease the risk of the patient removing the chest tube. Unlike the previous report, we found five serious complications with the use of SBWGTT (pulmonary contusions in three and lung trauma in two). One case died prior to surgery due to development of septic shock and the second required a lung lobectomy of the perforated lung lobe. Accidental lung perforation with the insertion of the SBWGTT is not previously reported in the previous literature in veterinary medicine (6, 7, 15). Accidental lung laceration associated with placement of thoracostomy tubes is reported in human medicine (18–20). In adults, lung perforation is reported with the use of trocar-type thoracostomy tubes (18–20) and in infants with pigtail-type thoracostomy tubes (21, 22) and ranges from 7 to 11% of cases (20, 23). Our large sample size may have permitted identification of this previously unreported serious but rare complication. The two cases of lung perforation reported in this study (one dog and one cat) were being treated for pyothorax, and the one taken to surgery for lung lobectomy had severe adhesions of the lung to the parietal pleura. Presence of adhesions close to the insertion site of the thoracostomy tube were found at autopsy in a case series of humans who had lung perforation with SBWGTT (19). Presence of adhesions secondary in inflammation in our pyothorax cases may have led to the lung lobe perforations in the cases reported here.

Identification of lung perforation by thoracostomy tubes using diagnostic imaging is challenging and often goes undetected on radiographs (18, 20, 24, 25). CT may provide better information as to the location of the chest tube and whether or not it is placed within the lung parenchyma (25). CT scans were done in only 43% of the cases reported in our study. Hence, our current findings may underestimate the true prevalence of intrapulmonary placement of SBWGTT as we relied on review of imaging and surgical reports to identify complications. Additionally, animals may not exhibit any worsening of respiratory distress or clinical signs of lung injury, so lung perforation may go undiagnosed. These findings are similar to findings in humans, in which patients do not always exhibit clinical signs associated with lung perforation, and the diagnosis of lung perforation is often made on autopsy (19, 21).

Pneumothorax was the most common complication reported with the use and insertion of SBWGTT overall. Our results concur with previously published reports in which no insertional complications were reported (6, 7, 15). Similarly, in human medicine, pneumothorax is associated with the placement and use of trocar-type thoracostomy tubes (26). Pneumothorax secondary to placement of SBWGTT could be due to accidental insertion of air within the thoracic cavity during placement and connection to the suction device. A small laceration to the lung parenchyma caused by the catheter or guide wire is also a possible explanation. Given the retrospective nature of our study, we were not able to determine which specific interventions were required to manage the pneumothoraces detected following placement of the chest tubes. In humans, no specific interventions other than clinical monitoring are recommended unless there is evidence of respiratory compromise or large volumes are being evacuated from the thoracotomy tube (24).

Infectious complications were the least frequent complications encountered in our study population. No empyema was reported. Development of empyema occurs in 1–25% of humans with various types of thoracostomy tubes (24). At our institution, SBWGTT are placed using an aseptic technique. Similarly, management of the thoracostomy tubes is done using sterile gloves, gauze, and syringes. Prophylactic antimicrobial therapy is not employed in patients with chest tubes other than cases diagnosed with pyothorax. Insertion site infections were reported in five cases overall. We were not able to identify the cause of the insertion site infections due to the retrospective nature of this study.

Unsurprisingly, complications were six times as likely (95% CI: 2.13–16.93) with two or more attempts at tube placement compared with tubes placed successfully on the first attempt. In humans, multimodal interventions, including use of checklists, ultrasound guidance for chest tube insertion, direct supervision of junior clinicians, and standardization of procedures are shown to successfully decrease complication rates for all types of thoracostomy tube placement (26, 27). Similar approaches in veterinary medicine could reduce the risk of complications by reducing the likelihood of multiple attempts at tube placement. Cats were not more likely to develop complications than dogs. This is consistent with the recent case series from Del Magno (7) in which the use of SBWGTT in feline pyothorax was shown to be effective and safe. Interestingly, logistic regression analysis showed that patients whose thoracostomy tubes were placed by ECC clinicians (who placed most of the chest tubes) did not have a decreased risk of complications. The positive effect of training, whether in a wet lab, use of simulators, or advancement through a residency program is well documented (28–30). Our study was possibly underpowered to detect a training or experience effect. Additionally, it is possible that, while non-ECC clinicians were the primary clinicians involved in thoracostomy tube placement, ECC clinicians may have assisted with the procedures. However, SBWGTT placed by ECC clinicians were more often accurately placed compared with those placed by non ECC clinicians. This could be an effect of placement technique and training. Due to its retrospective nature, we could not determine whether proper positioning assessed by radiograph corresponded to proper thoracostomy tube function. For instance, it is plausible that a more dorsal thoracostomy tube placement might allow for improved drainage of pneumothoraxes compared with the traditional cranio-ventral placement.

Use of sedation did not increase the risk of complications compared with the use of general anesthesia. This result is surprising as patients under general anesthesia are less likely to move from the discomfort of chest tube placement and/or suturing. General anesthesia may have been conducted in lieu of sedation for diagnostic imaging purposes, where SBWGTT were placed prior to CT or surgery. In addition, general anesthesia may have been performed based on the clinician's evaluation of the patient's stability and respiratory compromise, and perhaps more compromised patients who were less likely to move during the procedure were more likely to be sedated rather than anesthetized.

Our study had several limitations. Although a single author reviewed all medical records and diagnostic imaging, it is possible that some complications were not correctly reported, and therefore, our complication rate may be underestimated. Additionally, we were not able to review dwell time of thoracostomy tubes or efficiency of drainage or incidence of obstruction of the tubes. Numerous clinicians were involved in thoracostomy tube placement. Aside from the use of a sterile technique, there is not a standard operating procedure with regards to placement of SBWGTT in our institution. Specifically, the use of a small surgical blade to nick the skin at the insertion site or the use of the dilator is not standardized. Given that minor subcutaneous emphysema and swelling at the insertion site were common complications, standardization of the technique may have affected those results. At our institution, all small animal rotating interns undergo cadaver training on proper placement of SBWGTT.

In this cohort of dogs and cats, we found a 32% complication rate from placement of small-bore guide-wire thoracostomy tubes. Technical complications, such as soft tissue swelling at the insertion site, kinking of the thoracostomy tube, subcutaneous emphysema, and disconnection of the tube, were the most frequent. More importantly, insertional complications, such as small volume pneumothorax, lung perforation, and pneumomediastinum, occurred in 14% of cases with complications. Further studies are warranted to investigate interventions, such as the use of standardized techniques, including checklists, ultrasound guidance, and postprocedure bandaging to decrease complications and the associated morbidity in the future.

## Data Availability Statement

The original contributions presented in the study are included in the article/supplementary material, further inquiries can be directed to the corresponding author.

## Ethics Statement

Ethical review and approval was not required for the animal study because this was an observational, retrospective study. All animals were client-owned dogs and they had given the clinicians consent to treat and hospitalize. Written informed consent for participation was not obtained from the owners because this was an observational, retrospective study.

## Author Contributions

TB performed data extraction, analysis, and reviewed the manuscript. JM designed the study, performed data analysis, and wrote the manuscript. DF performed data analysis and reviewed the manuscript. JF, AB, LW, and MB performed data extraction and curation. All authors contributed to the article and approved the submitted version.

## Conflict of Interest

The authors declare that the research was conducted in the absence of any commercial or financial relationships that could be construed as a potential conflict of interest.

## Publisher's Note

All claims expressed in this article are solely those of the authors and do not necessarily represent those of their affiliated organizations, or those of the publisher, the editors and the reviewers. Any product that may be evaluated in this article, or claim that may be made by its manufacturer, is not guaranteed or endorsed by the publisher.

## Abbreviations

SBWGTT: Small-bore wire-guided thoracostomy tubes
ECC: Emergency and Critical Care
CT: Computed tomography
EMR: Electronic medical records
CI: confidence interval
FI: female intact
FS: female spayed
MC: male castrated
MI: male intact
HR: heart rate
RR: respiratory rate.
